# Stochastic gradient ascent learning with spike timing dependent plasticity

**DOI:** 10.1186/1471-2202-12-S1-P250

**Published:** 2011-07-18

**Authors:** Joana Vieira, Orlando Arévalo, Klaus Pawelzik

**Affiliations:** 1 Institute for Theoretical Physics, University of Bremen, Bremen, D-28359, Germany

## 

Stochastic gradient ascent learning exploits correlations of parameter variations with overall success of a system. This algorithmic idea has been related to neuronal network learning by postulating eligibility traces at synapses, which make them selectable for synaptic changes depending on later reward signals ([1] and [2]). Formalizations of the synaptic and neuronal dynamics supporting gradient ascent learning in terms of differential equations exhibit strong similarities with a recent formulation of spike timing dependent plasticity (STDP) [3] when it is combined with a reward signal. Here we present conditions under which reward modulated STDP is in fact guaranteed to maximize expected reward. We present numerical simulations underlining the relevance of realistic STDP models for reward dependent learning. In particular, we find that the nonlinear adaptation to pre- and post-synaptic activities of STDP [3] contributes to stable learning.

**Figure 1:**
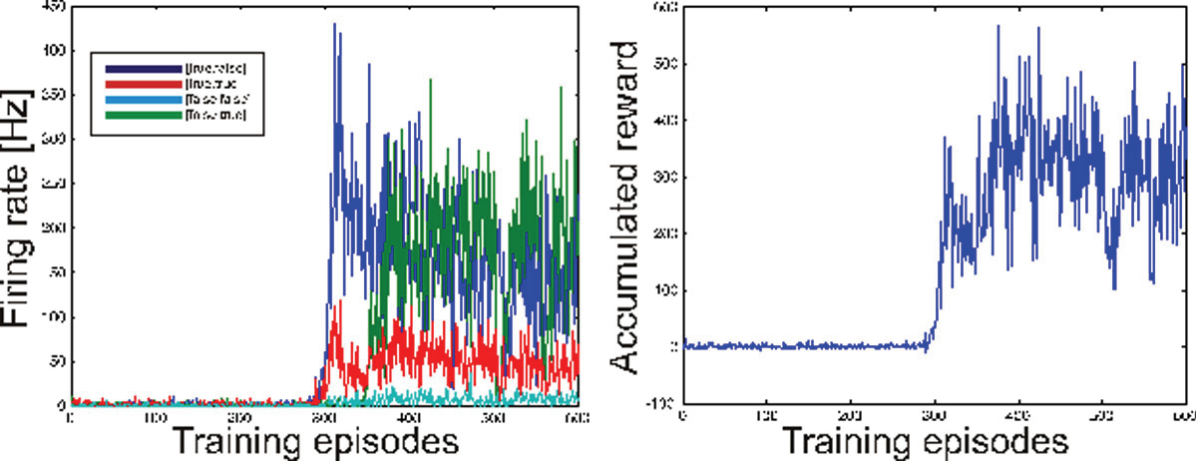
Learning the XOR function with a reward modulated STDP rule. Left: Output activity versus training episode in a feed forward network with Poisson-like neurons (2 input nodes, 10 hidden nodes and 1 output node). The output activity for the [true, false] and [false, true] inputs becomes stronger, while the output for the [true, true] and [false, false] inputs becomes weak after training. Right: Accumulated administered reward for the four input patterns versus training episode.
